# VALIDATION OF A NEW FUNCTIONAL COGNITION SCREENING MEASURE

**DOI:** 10.1093/geroni/igz038.1853

**Published:** 2019-11-08

**Authors:** Dorothy F Edwards, Muhammad O Al- heizan, Timothy Marks, Gordon M Giles

**Affiliations:** 1 University of Wisconsin Madison, Madison, Wisconsin, United States; 2 University of Wisconsin Madison, Madison Wisconsin, Wisconsin, United States; 3 Samuel Merritt University, Oakland, California, United States

## Abstract

Identification of cognitive deficits that contribute to functional dependence in acute and post-acute care settings is important. The Menu Task (MT) is a brief cognitive 12 item screening measure designed to identify older adults at risk for IADL impairment in community settings. The objective was to compare the psychometric properties of the Menu Task a series of neurocognitive and functional cognitive performance measures including the Brief Interview of Mental Status (BIMS), the Montreal Cognitive Assessment (MoCA) the Weekly Calendar Planning Activity (WCPA) and the Performance Assessment of Self-Care Skills (PASS) in a sample of 200 community dwelling older adults. Participants were administered the MT, BIMS, MoCA, WCPA, and the PASS checkbook and shopping tasks. ROC analysis identified an MT cut score, sensitivity and specificity. We computed Cronbach’s alpha, correlations among study measures and t-tests between groups impaired or unimpaired on the MT. Mean age of participants was 70.44(SD 8.3), the sample was predominately female ((76%), and white (81%). Mean MT score was 8.22 (SD 2.01) and the mean completion time was 185.5 sec (SD 108.11). The Menu Task has moderate internal consistency (α= 0.65). The AUC statistic was 0.83 with an optimal MT cut score of “7” and sensitivity of .90 and specificity of .70. Significant differences (p < 0.01) were observed between impaired and not impaired MT groups on BIMS, MoCA, WCPA, and PASS. The Menu Task has moderate to strong evidence supporting its psychometric properties and the value of screening for functional cognitive deficits.

